# Genotoxicity evaluation of Guibi-Tang extract using an *in vitro* bacterial reverse mutation assay, chromosome aberration assay, and *in vivo* micronucleus test

**DOI:** 10.1186/1472-6882-14-215

**Published:** 2014-07-01

**Authors:** Mee-Young Lee, Chang-Seob Seo, Ji-Young Kim, Hyeun-Kyoo Shin

**Affiliations:** 1 Herbal Medicine Formulation Research Group, Korea Institute of Oriental Medicine, 483 Expo-ro, Daejeon 305-811, Yusung-gu, Republic of Korea; 2 Division of Nonclinical Studies, Korea Institute of Toxicology, 19 Sinseongro, P.O. Box 123, Daejeon 305-343, Yuseong-gu, Republic of Korea

**Keywords:** Guibi-tang, Ames test, Chromosome aberration assay, Micronucleus, Genotoxic

## Abstract

**Background:**

Guibi-Tang is a traditional herbal prescription made from 12 different herbs that is used in the treatment of amnesia and poor memory.

**Methods:**

In the present study, we evaluated the acute oral toxicity and genotoxic potential of Guibi-Tang water extract (GBT) at doses up to 2000 μg/plate an using a bacterial reverse mutation test (Ames test) with *Salmonella typhimurium* strains TA100, TA1535, TA98, and TA1537, and *Escherichia coli* strain WP2uvrA. Acute toxicity and genotoxic potential were measured in the presence and absence of an exogenous source of metabolic activation, in an *in vitro* chromosome aberration assay with Chinese hamster lung (CHL) cells, and in an *in vivo* micronucleus test using ICR mice bone marrow as recommended by the Korean Food and Drug Administration. An acute oral toxicity test of GBT was performed in Sprague Dawley rats. The Ames test showed that GBT did not induce gene mutations in *S. typhimurium* or in *E. coli* in the presence or absence of S9 activation.

**Results:**

GBT did not significantly increase the number of structural aberrations in CHL cells with or without S9 activation. The oral administration of GBT at a dose of up to 2000 mg/kg caused no significant increase in the number of micronucleated polychromatic erythrocytes or in the mean ratio of polychromatic to total erythrocytes.

**Conclusions:**

However, as we did not identify the components of GBT responsible for these effects, other assays are needed to confirm its genotoxicity.

## Background

Traditional herbal medicines are selected to accentuate the therapeutic activity of their components while reducing the toxicity or side effects of compounds from other herbal species in the mixture [1].

The traditional herbal medicine Guibi-Tang (known as Qui-Pi-Tang in Chinese and Kihi-To in Japanese) is a mixture of 12 herbal preparations (Angelicae Gigantis Radix, Longan Arillus, Zizyphi Semen, Polygalae Radix, Ginseng Radix Alba, Astragali Radix, Atractylodis Rhizoma Alba, Hoelen Cum Radix, Aucklandiae Radix, Glycyrrhizae Radix, Zingiberis Rhizoma Crudus, and Zizyphi Fructus) [2]. This mixture has long been used to treat amnesia, poor memory or forgetfulness, fatigue, insomnia, anemia, palpitation, and neurosis.

Guibi-Tang has several pharmacological effects such as upregulation of choline acetyltransferase activity in rat embryo septal cultures [3]. Administration of Guibi-Tang for 3 months was reported to improve the scores on the Mini-Mental State Examination in 25 patients with senile dementia [4]. Although Guibi-Tang is used in the treatment of amnesia and its therapeutic efficacy has been studied, its toxicity remains unclear. Despite the popular use of centaury in traditional herbal medicine, no systematic evaluation of its genotoxic effects has been performed. Assessment of the genotoxic properties of folk medicine is important because damage to genetic material may lead to critical mutations and may thereby increase the risk of diseases including cancer [5]. The aim of the present study was to evaluate the safety of an aqueous extract of Guibi-Tang (GBT) and its potential genotoxicity. We assessed these properties using the standard battery of tests recommended by the Korea Food and Drug Administration: a bacterial reverse mutation test (Ames test), chromosome aberration test, and *in vivo* micronucleus test.

## Methods

### Preparation of GBT

The twelve crude herbs of GBT were purchased from Kwangmyungdang Medicinal herbs (Ulsan, Korea). The origin of each herbal medicine was taxonomically confirmed by Prof. Je Hyun Lee, Dongguk University, Gyeongju, Korea. Voucher specimens (2012-KE22-1 through KE22-12) have been deposited at the Herbal Medicine Formulation Research Group, Korea Institute of Oriental Medicine. A decoction of GBT was prepared in our laboratory from a mixture of chopped crude herbs (Table 1), and GBT was extracted in distilled water at 100°C for 2 h. The solution was evaporated to dryness and freeze-dried (yield: 22.9%). The extracted GBT powder was stored at 4°C.

**Table 1:** The combination of crude components of Guibi-Tang extract

**Herbal name**	**Scientific name**	**Amount (g)**	**Company of purchase**	**Source**
Angelicae Gigantis Radix	*Angelica gigas*	3.75	Kwangmyungdang Medicinal herbs	Yeongcheon, Korea
Longan Arillus	*Dimocarpus longan*	3.75	Kwangmyungdang Medicinal herbs	Vietnam
Ziayphi Semen	*Ziayphus jujuba*	3.75	Kwangmyungdang Medicinal herbs	China
Polygalae Radix	*Polygala tenuifolia*	3.75	Kwangmyungdang Medicinal herbs	China
Ginseng Radix Alba	*Panax ginseng*	3.75	Kwangmyungdang Medicinal herbs	Geumsan, Korea
Astragali Radix	*Astragalus membranaceus*	3.75	Kwangmyungdang Medicinal herbs	Jeongseon, Korea
Atractylodis Rhizoma Alba	*Atractylodes japonica*	3.75	Kwangmyungdang Medicinal herbs	China
Hoelen Cum Radix	*Poria cocos*	3.75	Kwangmyungdang Medicinal herbs	China
Aucklandiae Radix	*Aucklandia lappa*	1.875	Kwangmyungdang Medicinal herbs	China
Glycyrrhizse Radix	*Glycyrrhiza uralensis*	1.125	Kwangmyungdang Medicinal herbs	China
Zingiberis Rhizoma Crudus	*Zingiber officinale*	6.25	Kwangmyungdang Medicinal herbs	Yeongcheon, Korea
Zizyphi Fructus	*Zizyphus jujuba*	3.75	Kwangmyungdang Medicinal herbs	Yeongcheon, Korea
Total		35.625		

### HPLC analysis of GBT

HPLC simultaneous determination was performed using a Shimadzu LC-20A HPLC system (Shimadzu Co., Kyoto, Japan), consisting of LC-20AT pump, DGU-20A_3_ online degasser, SPD-M20A detector, SIL-20 AC auto-sampler, and CTO-20A column oven. The data processor employed LC solution software (Version 1.24). The separation of three compounds was conduct using a Gemini C_18_ (250 × 4.6 mm; 5 μm, Phenomenex, Torrance, CA, USA) and column oven temperature was maintained at 40°C. The mobile phases were composed of 1.0% (v/v) aqueous acetic acid (A) and 1.0% (v/v) acetic acid in acetonitrile (B). The gradient condition was as follows: 0–30 min, 10–70% B; 30–35 min, 70–100% B; 35–40 min, 100–100% B; 40–45 min, 100–10% B; 45–60 min, 10–10% B. The analysis was conduct at a flow-rate of 1.0 mL/min with PDA detection at 254 nm, 280 nm, and 330 nm. The injection volume was 10 μL.

The three compounds in GBT were well separated using the developed HPLC method. The retention times of the three major components under the optimized HPLC assay were 14.23, 15.16, and 29.07 min for liquiritin, nodakenin, and glycyrrhizin, respectively. The amount of three quantify compounds in GBT included 0.70 ± 0.01 mg/g (liquiritin), 0.03 ± 0.01 mg/g (glycyrrhizin), and 1.43 ± 0.01 mg/g (nodakenin), which are components of Glycyrrhizae Radix et Rhizoma and Angelicae Gigantis Radix, respectively.

### Acute oral toxicity test

To test the acute oral toxicity, specific pathogen-free Sprague Dawley rats of both sexes were obtained at 5 weeks of age from Orient Bio Co., Ltd. (Seongnam, Korea) and used after 1 week of quarantine and acclimatization. This study was approved by the Korea Institute of Oriental Medicine Institutional Animal Care and Use Committee, was performed at the Korea Institute of Toxicology (Daejeon, Republic of Korea), and was conducted according to the guidelines of the Institutional Animal Care and Use Committee in the Korea Institute of Toxicology, which is accredited by AAALAC International (1998) under the GLP Regulations for Nonclinical Laboratory Studies. A preliminary study showed that a single oral administration of GBT did not induce any toxic effect at dose levels of 0 and 2000 mg/kg/day. Based on these results, a dose of 2000 mg/kg/day was selected as the toxicological limited dose recommended by the OECD guidelines. Healthy male and female rats were assigned to groups of five rats of each sex. GBT was suspended in distilled water, and the volume for application of a dose of 10 mL/kg body weight was calculated. The vehicle control rats received an equivalent volume of distilled water only. All animals were observed, and mortality, clinical signs, body weight changes, and gross findings were recorded for 14 days.

### Preparation of the S9 mixture

The two *in vitro* genotoxicity tests were conducted in compliance with the OECD guidelines for the testing of chemicals (July 21, 1997) in the publications TG No. 473 ‘In vitro Mammalian Chromosome Aberration Test’ and TG No. 471 ‘Bacterial Reverse Mutation Test’ with and without metabolic activation (OECD guidelines TG 473 and TG 471, 1997).

Rat liver microsomal enzyme (S9) prepared male Sprague–Dawley rats induced with Aroclor 1254, was obtained from Molecular Toxicology Inc. (Boon, NC, USA). Before the experiment, an appropriate quantity of S9 supernatant was thawed and mixed with S9 cofactor solution. The amount of S9 supernatant was 10% v/v in the S9 mixture. Cofactors were added to the S9 mixture to reach the final concentrations of 8 mM MgCl_2_, 33 mM KCl, 5 mM glucose-6-phosphate, and 5 mM NADP in the S9 mixture.

### Bacterial reverse mutation assay (Ames test)

The experimental methods used in the study were based on the published reports by Maron and Ames [6] with minor modifications. *Salmonella typhimurium* strains TA98 and TA1537 (to detect frame-shift mutagens), TA100, and TA1535, and *Escherichia coli* strain WP2uvrA (to detect base pair-substitution mutagens) were obtained from Molecular Toxicology Inc. (Boone, NC, USA) and were used as the tester strains. To evaluate the toxicity and solubility (precipitation) of GBT, a pilot experiment was performed with all bacterial strains. GBT was dissolved in distilled water. The positive control factors were dissolved in either distilled water or DMSO and stored at -20°C; these positive control factors were 2-nitrofluorene (2-NF), 2-aminoanthracene (2-AA), 9-aminoacridine (9-AA), 4-nitroquinoline X-oxide (4NQO), and benzo (a) pyrene (BP). A dose range-finding test was performed to determine the highest concentration for the present study, which was performed with the five tester strains at concentrations of 625, 1250, 2500, and 5,000 μg/plate with and without the S9 mixture. The number of revertant colonies did not increase to more than twice the value observed in the controls for any of the tester stains. However, there were increased numbers of revertant colonies of TA1535 with the S9 mixture. Based on these results, a dose of 5,000 μg/plate was selected as the maximum dose.

Briefly, various concentrations of GBT were incubated with the tester strains at 37°C for 48 h in the presence or absence of metabolic activation by the S9 mixture along with vehicle and positive controls containing the following combinations of substances and doses: 2-AA at 2 μg/plate vs. TA1535 with or without the S9 mixture and at 4 μg/plate vs. WP2uvrA with the S9 mixture; 9-AA at 50 μg/plate vs. TA1537 without the S9 mixture; BP at 2 μg/plate vs. TA98 with or without the S9 mixture and vs. TA100 and TA1537 with the S9 mixture); 2-NF at 2 μg/plate vs. TA98 without the S9 mixture; 4NQO at 0.5 μg/plate vs. WP2uvrA without the S9 mixture; and sodium azide at 0.5 μg/plate vs. TA100 and TA1535 without the S9 mixture. Each concentration of GBT was tested in triplicate. A result was deemed positive if there was a concentration-related increase over the range tested and/or a reproducible increase at one or more concentrations in the number of revertant colonies per plate in at least one strain with or without the S9 mixture. An antibacterial effect (cytotoxicity) was defined as a clearing or diminution of the background lawn, the appearance of microcolonies, and/or a decrease of > 50% in the number of colonies compared with the relevant vehicle control.

### Chromosome aberration test

Chinese hamster lung (CHL) cells were obtained from American Type Culture Collection (Manassas, VA, USA) in 2004. The cells were thawed in culture medium and then grown for more than 7 days as a monolayer. Cells were cultured in reconstituted MEM (Gibco-Invitrogen, USA) supplemented with 2.2 g of sodium bicarbonate, 292 mg of l-glutamine, streptomycin sulfate (100 μg/mL), penicillin G · Na (10^5^ units), and 10% (v/v) fetal bovine serum (FBS; Gibco-Invitrogen, USA) per liter. The cultures were incubated at 37°C in a humidified atmosphere with 1.5% CO_2_. A preliminary dose range-finding study was performed to determine the highest concentration for this study. Using the results from the dose range-finding study, the dose range for the present study was designed to quantify the solubility and cytotoxicity of GBT. Ethyl methanesulfonate (EMS) was used as a positive control substance without metabolic activation and cyclophosphamide (CPA) with metabolic activation.

Cells were trypsinized and counted, and the relative cell count (RCC) was calculated. The cells were centrifuged at ~1000 rpm for 5 min and resuspended in 5 mL of 75 mM KCl solution. After 10 min at room temperature, 5 mL of fixative (methanol:glacial acetic acid = 3:1 v/v) was added to the cell suspension, and the suspension was refrigerated for ~20 min. The fixative was changed twice by centrifugation at ~1500 rpm for 5 min. Two slides were prepared from each fixed-cell suspension. The slides were air-dried, stained with 3% Giemsa solution, washed in tap water and distilled water, dried, and mounted in DPX (Fluka) for chromosome aberration scoring.

Chromosome aberrations were identified morphologically according to the principles described in the ‘Atlas of chromosome aberration by chemicals’ (JEMS-MMS, 1988). Cells with more than four of the same type of aberration were scored as multiple aberrations. Any metaphase with one or more aberrations, regardless of the type, was classified as an aberration metaphase. Slides were scanned systemically, and each set of metaphases was examined at 1000× magnification. Structural chromosome aberrations were evaluated in 100 well-spread metaphases, each containing 23 to 27 chromosomes. The microscopic stage coordinates and each type and number of aberration were recorded for each aberrant metaphase. The results are expressed as the number of findings per 100 metaphases. Regardless of the presence of aberrations, an additional 100 metaphases were examined to determine the frequency of diploidy (DP), polyploidy (PP, > 37 chromosomes), and endoreduplication (ER).

### *In vivo* micronucleus test

The preliminary study showed that oral administration of GBT at a dose of 2000 mg/kg did not induce any toxic effect. The highest dose was determined based on the dose range-finding study, and 2000 mg/kg, which was the limit dose for treatment up to 14 days according to the OECD guidelines, was selected as the maximum dose. Specific pathogen-free male CrljOri:CD1 (ICR) mice weighing 27.2–30.0 g were obtained from Orient Bio Co., Ltd. (Seongnam, Korea) at 6 weeks of age. Mice were used in experiments after 1 week of quarantine and acclimatization. This study was reviewed and assessed by the IACUC of the Korea Institute of Toxicology. All animals were cared for in accordance with the principles outlined in the NIH Guide for the Care and Use of Laboratory Animals (AAALAC International accredited in 1998). GBT was administered once a day for 2 days by gavage to male ICR mice. The positive control was CPA monohydrate in normal saline (10 mL/kg), which was given at a dose of 70 mg/kg i.p. just before the dose on the second day of dosing [7].

Animals were sacrificed by CO_2_ gas inhalation at ~24 h after the final administration, and bone marrow preparations were made using the method of Schmid [8].

Two slides of the cell suspension from each animal were made. Small round or oval bodies, measuring about 1/5 to 1/20 the diameter of a polychromatic erythrocyte (PCE), were counted as micronuclei. The same observer scored a total of 2000 PCEs per animal to determine the frequency of micronucleated polychromatic erythrocytes (MNPCEs). The ratio of PCEs to normochromatic erythrocytes (NCE) [PCE/(PCE + NCE) ratio] was calculated by counting 500 cells. The mortality and external appearance of animals were checked and recorded once a day during the study period, and these observations were made three times after the final administration on the final dosing day. Body weight was measured on the days of reception, grouping, dosing, and autopsy.

### Statistical analyses

The statistical analyses used for the study were selected based on the methods used in published reports [9] using SAS software (version 9.1.3, SAS Institute Inc., Cary, NC, USA). Each metaphase was classified as a normal metaphase or aberrant metaphase with one or more aberrations, and the frequency of aberrant metaphase was analyzed statistically. The numerical aberrations were classified into DP, PP, and ER, and the frequencies of PP + ER were analyzed. The χ^2^ test and Fisher’s exact test were performed to compare the vehicle control and GBT-treated groups [10]. Fisher’s exact test was used to compare the vehicle and positive control groups. Differences were regarded as significant at *P* < 0.05.

Statistical evaluation of the *in vivo* micronucleus results was performed using the method of Lovell et al. [11] with minor modification. Data with heterogeneous variances were analyzed using Kruskal–Wallis analysis of variance followed by multiple comparisons using Dunnett’s test [12]. The significance was accepted when all of the PCE/(PCE + NCE) ratios were > 0.1. The result was judged as positive when there was a significant and dose-related increase or a reproducible increase in the frequency of MNPCEs or aberrant metaphases at one or more dose levels. Differences were regarded as significant at *P* < 0.05.

## Results

### Acute oral toxicity test

**Figure 1:**
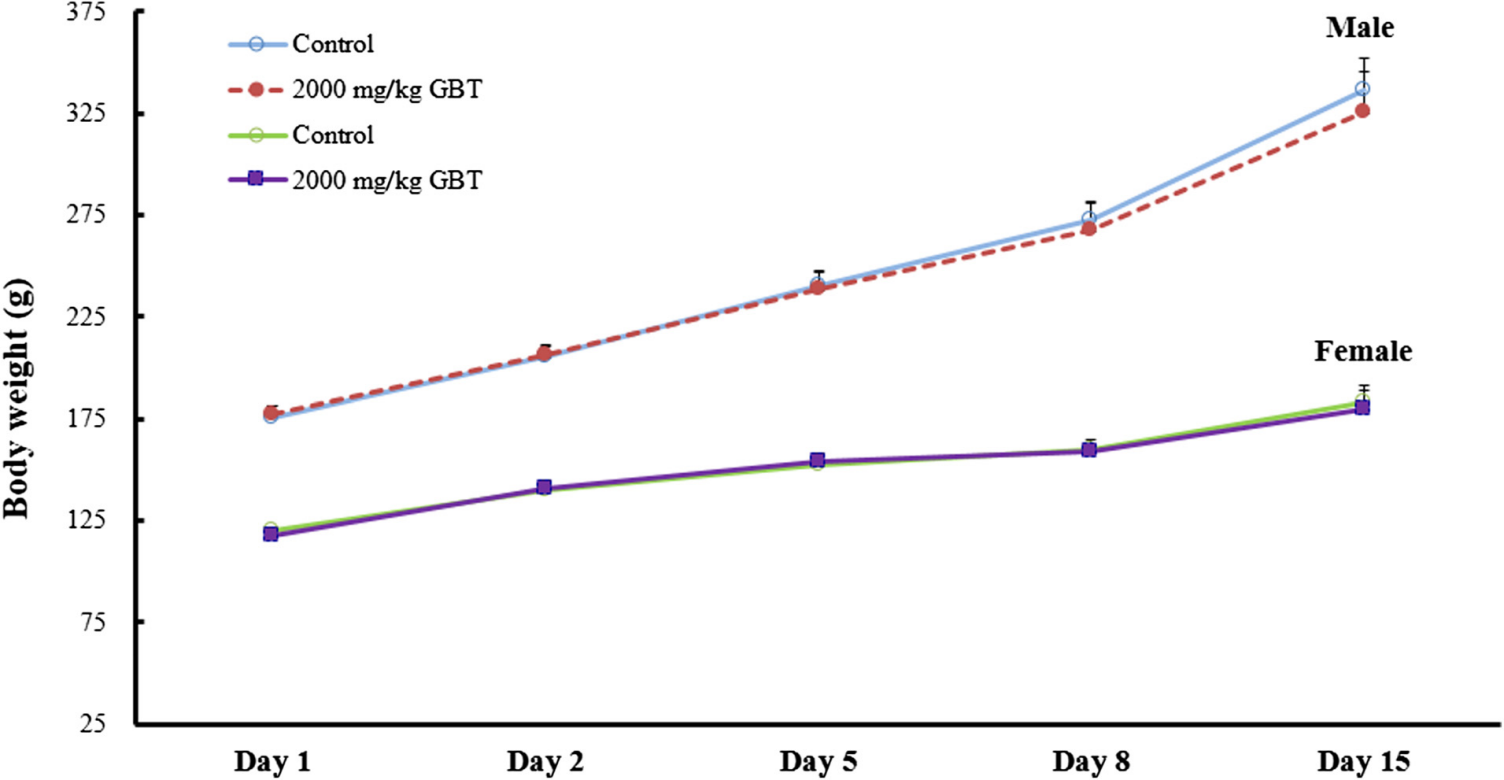
**Mean body weight changes after administrated with GBT at dose levels of 0 (vehicle) and 2000 mg/kg/day.** Values are presented as mean ± SD.

No mortality or clinical symptoms of toxicity were observed in males or females in any group during the observation period of 14 days. The body weight changes are summarized in Figure 1. In both sexes, the changes in body weight did not differ significantly between the group treated with 2000 mg/kg/day of GBT and the vehicle control group. At the time of the scheduled autopsy, there were no abnormal observations of the internal organs including the lung, heart, thymus, stomach, liver, adrenals, and spleen in the males or females given 2000 mg/kg/day of GBT.

### Bacterial reverse mutation assay (Ames test)

**Figure 2:**
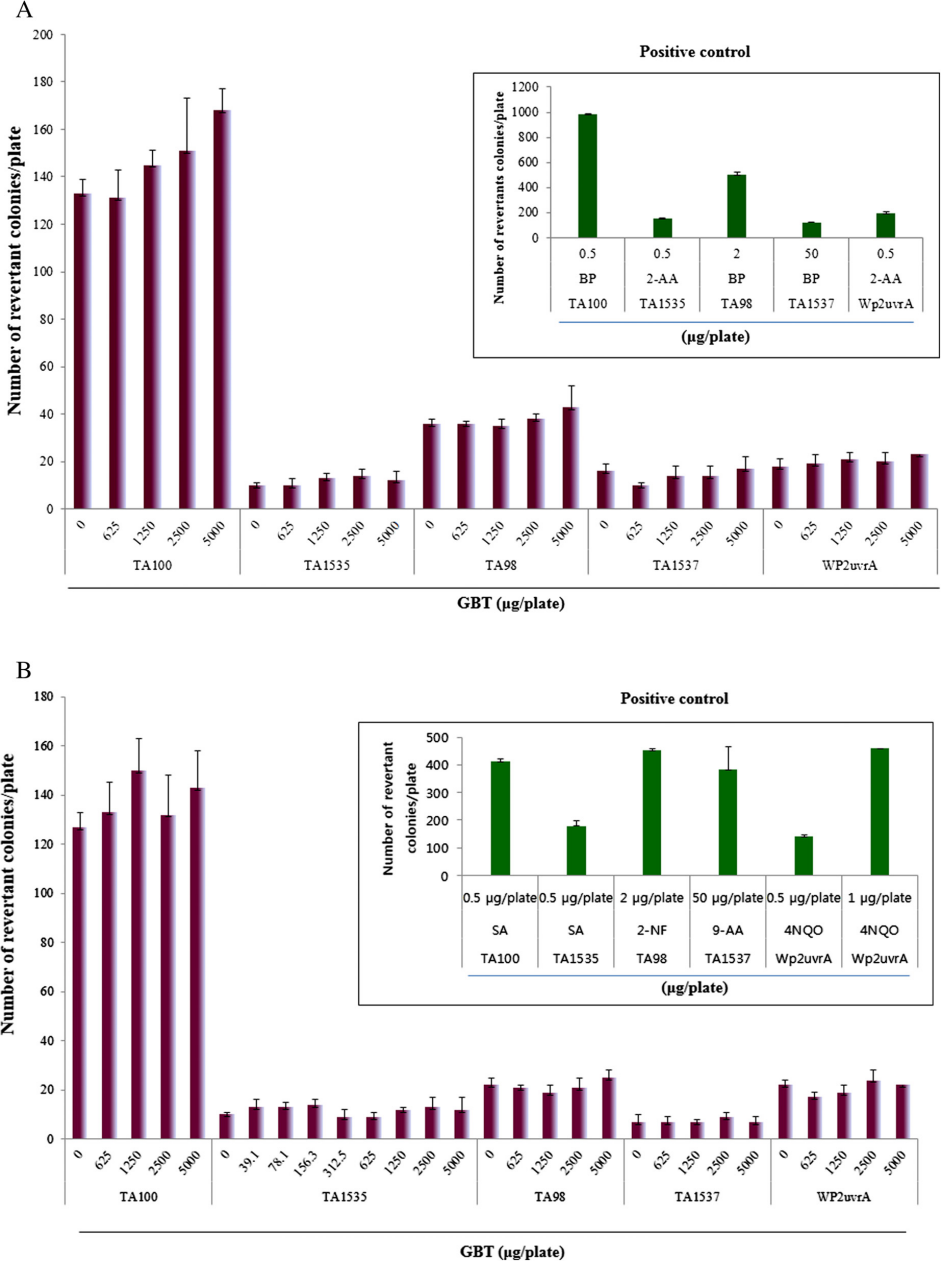
Effect of GBT on bacterial reverse mutation assay (Ames test) (A) with (+S9 mix) and (B) without (-S9 mix) metabolic activation.

No positive mutagenic response was observed in any of the *S. typhimurium* or *E. coli* strains tested compared with the concurrent vehicle control groups regardless of the presence (Figure 2A) or absence (Figure 2B) of the S9 mixture. The positive controls showed significantly increased numbers of revertant colonies, indicating that the assay was valid.

### Chromosome aberration tests

According to our preliminary study (data not shown), GBT neither inhibited cell growth nor killed CHL cells. We determined the concentration range that was most compatible with a good cell-proliferating ability and that produced a sufficient number of metaphases for the confirmatory assay. Therefore, we used 5000 μg/mL as the highest exposure level and serial dilutions for further dose–response tests.

There were no observable increases in the frequency of metaphases with aberrant chromosomes at 6 h or 22 h with or without the S9 mixture in the GBT-treated group compared with the vehicle control group (Table 2).

**Table 2:** Chromosome aberration assay and relative cell counts of Guibi-Tang (GBT) extract

**Nominal conc. of GBT (μg/mL)**	**S9 mix**	**Time hours a)**	**Mean total aberrant metaphases**	**Mean total aberrations**	**Mean of PP+ER**	**Relative cell counts (%)**
6 h treatment (+S9)						
0	+	6-18	0.5/0.5	1.0/1.0	3.0 + 0.0	100
1250	+	6-18	1.0/1.0	2.0/2.0	0.5 + 0.0	96
2500	+	6-18	1.5/1.0	2.0/1.5	1.0 + 0.0	96
5000	+	6-18	2.0/1.5	2.5/2.0	2.0 + 0.0	85
CPA 6	+	6-18	30.0/29.5**	44.0/43.5	1.0 + 0.0	62
6 h treatment (-S9)						
0	-	6-18	0.0/0.0	0.0/0.0	1.0 + 0.0	100
1250	-	6-18	0.0/0.0	0.0/0.0	1.0 + 0.0	94
2500	-	6-18	2.5/2.5	2.5/2.5	1.0 + 0.0	92
5000	-	6-18	0.5/0.5	1.0/1.0	2.0 + 0.0	83
EMS 800	-	6-18	22.5/22.0**	34.0/33.0	0.5 + 0.0	63
22 h treatment (-S9)						
0	-	22-2	0.0/0.0	0.0/0.0	1.0 + 0.0	100
1250	-	22-2	0.0/0.0	0.0/0.0	1.5 + 0.0	94
2500	-	22-2	0.5/0.5	1.0/1.0	0.5 + 0.0	89
5000	-	22-2	2.0/1.5	3.5/3.0	1.0 + 0.0	80
EMS 600	-	22-2	31.5/31.5**	46.0/46.0	1.0 + 0.0	54

**Significantly different from the control at P<0.01.

a) Treatment time-recovery time.

### Micronucleus test

No abnormal changes were observed in the general appearance or body weight between the first and final administrations in the vehicle control group, positive control group, or the groups treated with 500, 1000, or 2000 mg/kg/day of GBT (Table 3). The number of MNPCEs/2000 PCEs and PCE/(PCE + NCE) did not increase significantly in the groups treated with GBT at 500, 1000, or 2000 mg/kg/day (Table 4). There was significant increase in the number of MNPCE/2000PCEs in the positive control group, indicating that the present study was preformed under acceptable experimental conditions.

**Table 3:** Body weight changes of Micronucleus test in mice following administration of Guibi-Tang (GBT) extract

**Group**	**Vehicle control**	**GBT**	**GBT**	**GBT**	**Positive control**
**Dose (mg/kg)**	**0**	**500**	**1000**	**2000**	**70**
Day 1 (Mean ± S.D)	34.6 ± 0.79	34.7 ± 1.34	34.8 ± 1.14	35.1 ± 0.57	35.5 ± 0.63
Day 2 (Mean ± S.D)	34.9 ± 1.42	35.2 ± 1.21	35.2 ± 1.19	35.3 ± 0.96	35.3 ± 0.40
Day 3 (Mean ± S.D)	34.8 ± 1.04	35.5 ± 1.11	35.2 ± 0.72	35.0 ± 0.69	34.5 ± 0.67

**Table 4:** Micronucleus test in mice following a single oral dose of Guibi-Tang (GBT) extract

**Group**	**Vehicle control**	**GBT**	**GBT**	**GBT**	**Positive control (cyclophosphamide)**
**Dose (mg/kg)**	**0**	**500**	**1000**	**2000**	**70**
MNPCE/2000PCEs (Mean ± S.D)	0.33 ± 0.58	1.67 ± 1.15	1.00 ± 1.00	0.33 ± 0.58	62.33* ± 10.21
PCE/(PCE+NCE) (Mean ± S.D)	0.48 ± 0.05	0.53 ± 0.07	0.50 ± 0.08	0.53 ± 0.05	0.46 ± 0.03
Number of animals	3	3	3	3	3

*MNPCE*: PCE with one or more micronuclei; *PCE*: Polychromatic erythrocyte; *NCE*: Normochromatic erythrocyte.

*Significantly different from the control at *p*<0.05.

## Discussion

Despite the frequent use of medicinal plants, few scientific studies have been undertaken to determine the safety of traditional medicinal herbs. To determine the safety of medicines and plant products intended for human consumption, systematic toxicological studies must be performed using various experimental models to predict the toxicity and to set the criteria for selecting a safe dose in humans. Genotoxicity tests have been used mainly for the prediction of genotoxicity and carcinogenicity of chemicals because compounds that are positive in these tests have carcinogenic and/or mutagenic potential in humans [13-15]. However, most commonly used herbal formulas have no indications of quality, safety, and efficacy. Guibi-tang is a traditional Korean medicinal formula that has been used to treat amnesia and memory for several hundred years. However, its toxicity study remains unclear. Therefore, in the present study we evaluate the potential genotoxicity of GBT, we performed tests to detect chromosome aberrations in CHL cells, a bacterial reverse mutation test using the *S. typhimurium/E. coli* incorporation assay (Ames test), and an *in vivo* micronucleus test.

The Ames test uses amino acid-requiring strains of *S. typhimurium* and *E. coli* to detect point mutations involving substitution, addition, or deletions of one or more DNA base pairs [5]. We found no positive mutagenic responses to GBT in any of the tester strains compared with the concurrent vehicle control groups both with and without application of the S9 mixture. This is a widely accepted short-term assay to identify substances that can produce genetic damage leading to gene mutations [16].

Chromosome aberrations are the classical genotoxic response to tumor initiation and development processes [17,18]. The purpose of the *in vitro* chromosome aberration test is to identify agents that cause structural chromosome aberrations in cultured mammalian cells [19,20]. CHL cells [21] were selected as the test system because they are sensitive to mutagens, their low chromosome number facilitates scoring, and they can be used for repeated measurements. In the present study, the results of the chromosome aberration assay demonstrated clearly that there were no significant increases in the number of metaphases with structural aberrations at any dose of GBT in the presence or absence of the metabolic activation system in CHL cells.

The micronucleus test detects mutagenic substances, thus altering the equitable distribution of chromosomes during cell division [22]. The micronucleus assay in male mice showed no significant increases in the frequency of micronuclei at any dose of GBT (500, 1000, or 2000 mg/kg/day) compared with the vehicle control group.

## Conclusion

In conclusion, GBT had no genotoxic effects in various tests involving bacteria and mammals, suggesting that GBT may not cause mutations in bacterial systems or chromosome or DNA damage *in vitro* and *in vivo*. Further detailed experiments are needed to identify whether GBT contains any genotoxic components and, if so, the underlying mechanism(s).

## Competing interests

The authors declare that they have no competing interests.

## Authors’ contributions

MYL and HKS participated in the design of the study data analyses and manuscript preparation. MYL, CSS, JYK and HKS conducted the assays and analyses. All authors read and approved the final manuscript.

## Pre-publication history

The pre-publication history for this paper can be accessed here:

http://www.biomedcentral.com/1472-6882/14/215/prepub
